# Zambia field epidemiology training program: strengthening health security through workforce development

**DOI:** 10.11604/pamj.2020.36.323.20917

**Published:** 2020-08-21

**Authors:** Ramya Kumar, Ernest Kateule, Nyambe Sinyange, Warren Malambo, Shadrick Kayeye, Elizabeth Chizema, Gershom Chongwe, Patrick Minor, Muzala Kapina, Henry C Baggett, Ellen Yard, Victor Mukonka

**Affiliations:** 1 Zambia AIDS Related Tuberculosis Project, Lusaka, Zambia,; 2 Zambia Field Epidemiology Training Program, Ministry of Health, Lusaka, Zambia,; 3 Zambia National Public Health Institute, Lusaka, Zambia,; 4 U.S. Centers for Disease Control and Prevention (CDC), Lusaka, Zambia,; 5 ICAP Columbia University, Lusaka, Zambia,; 6 National Malaria Elimination Centre, Ministry of Health, Lusaka, Zambia,; 7 University of Zambia, School of Public Health, Lusaka, Zambia,; 8 Division of Global HIV & TB, CDC, Atlanta, Georgia, USA,; 9 Division of Global Health Protection, CDC, Atlanta, Georgia, USA

**Keywords:** Sub-Saharan Africa, outbreak investigation, public health surveillance, National Public Health Institute

## Abstract

The Zambia Field Epidemiology Training Program (ZFETP) was established by the Ministry of Health (MoH) during 2014, in order to increase the number of trained field epidemiologists who can investigate outbreaks, strengthen disease surveillance, and support data-driven decision making. We describe the ZFETP´s approach to public health workforce development and health security strengthening, key milestones five years after program launch, and recommendations to ensure program sustainability. Program description: ZFETP was established as a tripartite arrangement between the Zambia MoH, the University of Zambia School of Public Health, and the U.S. Centers for Disease Control and Prevention. The program runs two tiers: Advanced and Frontline. To date, ZFETP has enrolled three FETP-Advanced cohorts (training 24 residents) and four Frontline cohorts (training 71 trainees). In 2016, ZFETP moved organizationally to the newly established Zambia National Public Health Institute (ZNPHI). This re-positioning raised the program´s profile by providing residents with increased opportunities to lead high-profile outbreak investigations and analyze national surveillance data-achievements that were recognized on a national stage. These successes attracted investment from the Government of Republic of Zambia (GRZ) and donors, thus accelerating field epidemiology workforce capacity development in Zambia. In its first five years, ZFETP achieved early success due in part to commitment from GRZ, and organizational positioning within the newly formed ZNPHI, which have catalyzed ZFETP´s institutionalization. During the next five years, ZFETP seeks to sustain this momentum by expanding training of both tiers, in order to accelerate the professional development of field epidemiologists at all levels of the public health system.

## Introduction

Every nation needs a public health system that can detect and respond to domestic and global public health threats in a timely way [1-3]. Many nations in sub-Saharan Africa face challenges, such as a shortage of adequately trained personnel in the public health sector including a lack of trained field epidemiologists [4]. Field epidemiologists (i.e. disease detectives) collect, analyze, and interpret data for evidence-based decision-making. Epidemiologists bolster public health surveillance systems, which can help a country better understand their burden of disease and allocate limited resources. Epidemiologists quickly detect and respond to disease outbreaks, and provide recommendations for evidence-based interventions. Their efforts can reduce morbidity and mortality and reduce the risk of diseases spreading across borders [5]. These functions are even stronger when networked within an organized public health system, such as a national public health institute (NPHI) [6, 7].

The World Health Organisation (WHO) recognizes a strong public health workforce as critical to compliance with the 2005 International Health Regulations [1]. The IHR Monitoring and Evaluation Framework recommends a target of one trained field epidemiologist per 200,000 population [8]. According to this WHO standard, Zambia-a country of 16.8 million people would need to employ 84 field epidemiologists. To this end, the IHR´s Joint External Evaluation (JEE) tool recommends that countries establish field epidemiology training programs [9].

Despite the threat of epidemic-prone diseases such as measles, typhoid, cholera, and avian influenza, in 2014 the Zambian Ministry of Health (MoH) employed only two epidemiologists [10]; both were stationed at MoH headquarters in Lusaka. Outbreaks were typically investigated in an ad-hoc manner, and although outbreak investigations were summarized in reports, these reports were not stored in an easily-accessible public repository, or published in the literature.

As part of a multi-faceted approach to strengthen health security in Zambia, the MoH established its Field Epidemiology Training Program (FETP) in 2014. This program was created in order to increase the number of trained field epidemiologists who could investigate public health events, strengthen routine disease surveillance, and analyze data to drive evidence-based decision making. This paper describes the development of Zambia´s FETP as a strategy to strengthen health security, while highlighting solutions to start-up challenges, and making recommendations to ensure program sustainability five years after the program launch (September 2014 - October 2019).

## Project evaluation

**ZFETP description:** ZFETP was established by the Zambia MoH in 2014 as a tripartite arrangement: 1) the MoH launched the program within the Department of Disease Surveillance and Response and provided the trainees with field placement sites; 2) the University of Zambia (UNZA) School of Public Health cultivated epidemiologic and biostatistical knowledge through didactic coursework; and 3) the U.S. Centers for Disease Control and Prevention (CDC) provided funding, technical guidance and mentorship. From program inception, U.S. CDC has provided technical and administrative support via a full-time in-country Resident Advisor, public health specialists, and masters-level epidemiology fellows. The program is currently physically housed within the Zambia National Public Health Institute (ZNPHI) and is advised by a Steering Committee comprised of representatives from MoH, UNZA School of Public Health, U.S. CDC, National Health Research Authority (a national regulatory body for research), and the UNZA School of Veterinary Medicine. The ZFETP aims to: 1) develop capacity to train public health professionals in applied or field epidemiology; 2) provide epidemiological services to strengthen health security at national, provincial, district and local levels; and 3) reduce the burden of priority public health problems through strengthened epidemiology capacity and the service provided by FETP trainees [11]. ZFETP conducts two training tiers (FETP - Advanced and FETP - Frontline) to strengthen surveillance and epidemiological skills at national and subnational levels of the public health system.

**Figure 1:**
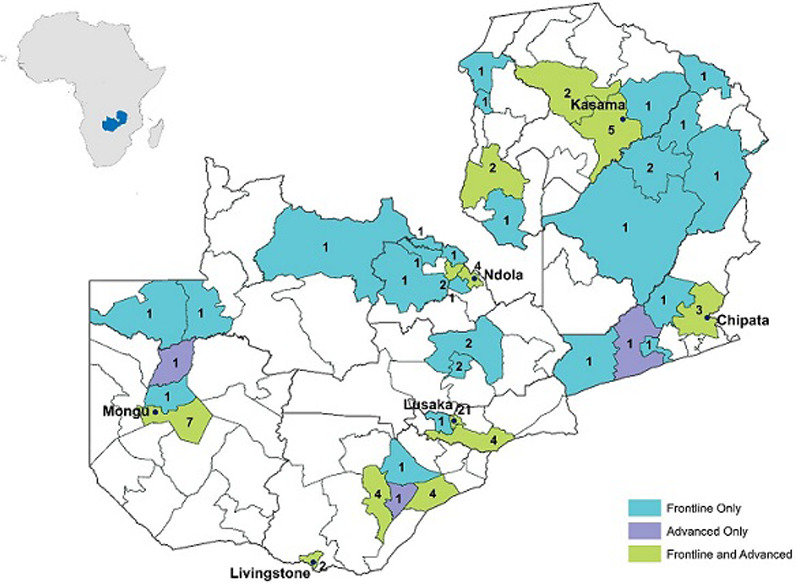
districts with advanced and frontline ZFETP graduates, Zambia, 2019

**FETP-Advanced:** ZFETP-Advanced is a two-year, full-time program that began in 2014 and consists of approximately 30% classroom training and 70% fieldwork. Trainees (i.e. residents) spend approximately seven months in didactic coursework at UNZASOPH (6 months of coursework and 1 month of examinations), followed by 17 months in field placements. During the field placements, the residents receive hands-on training and experience in evaluating public health surveillance systems, investigating disease outbreaks, and conducting hypothesis-driven epidemiologic analyses that address priority public health issues at local or national levels. Successful residents receive a Masters of Science (MSc) in Epidemiology. ZFETP-Advanced builds competencies in public health surveillance, outbreak response, public health research, and scientific communication, with the expectation that graduates will assume public health leadership positions, and serve as mentors to future trainees and junior field epidemiologists [12-14]. Currently, only MoH employees are eligible for ZFETP - Advanced, and employees must take study leave from their full-time positions. MoH continues to pay their salaries during training, and residents receive support that includes housing, university tuition, equipment (e.g. laptops, cameras), and internet bundles. In return, graduates incur a two-year service obligation to the Government of the Republic of Zambia (GRZ) after completing training. From September 2014 through 2018, ZFETP has enrolled three Advanced cohorts, graduating a total of 14 residents (Table 1); the majority of graduates work in the capital city of Lusaka (Figure 1). An additional 10 residents are scheduled to graduate in September 2020. ZFETP enrolls health professionals from various educational backgrounds beyond medicine, such as laboratory sciences, public health, population studies, epidemiology, and nursing (Table 1). This approach promotes the exchange of knowledge and skills, and contributes to a multi-disciplinary response to public health events.

**Table 1:** demographic characteristics of enrolled Zambia FETP residents and trainees, 2014 - 2019 (N = 95) †

	Advanced			Frontline			
Cohort number (Year Enrolled)	1 (2014)	2 (2016)	3 (2019)	#1 (2015)	#2 (2017)	#3 (2018)	#4 (2019)
	N=6	N=8	N=10	N=13	N=16	N=21	N=21
	**n/N (%)**	**n/N (%)**	**n/N (%)**	**n/N (%)**	**n/N (%)**	**n/N (%)**	**n/N (%)**
**Sex**							
Male	5 (83%)	5 (62%)	7 (70%)	10 (76%)	11 (69%)	11 (70%)	15 (62%)
Professional Background*							
Medicine**	3 (50%)	2 (25%)	4 (40%)	11 (79%)	6 (38%)	1 (5%)	4 (14%)
Public Health	1(17%)	4 (50%)	6 (60%)	2 (14%)	10 (62%)	14 (90%)	15 (62%)
Laboratory Sciences	0	1(12%)	0	1 (7%)	0	0	2 (9%)
Other ***	2(33%)	1(12%)	0	0	0	1 (5%)	0

* First degrees at the time of enrollment; **Bachelor of Medicine (i.e. MBBS, MB ChB); *** Includes Population Studies, Epidemiology, and Nursing; † of the 95 residents and trainees, 8 were enrolled in both Frontline and Advanced programs

### ZFETP-Frontline

Zambia´s FETP-Frontline is an in-service program launched in 2015. It is primarily focused on strengthening surveillance and enhancing early outbreak detection at sub-national levels of the public health system. Similar to ZFETP-Advanced, ZFETP-Frontline trainees must be MoH employees. Frontline accepts surveillance officers and health directors from districts and provinces across the country. The target is to train at least 1 surveillance officer in each district by 2026. To date, the program has enrolled 4 cohorts and graduated 71 trainees from November 2015 through 2018 (Table 1); 41 (35%) out of 117 districts have at least one trained Frontline resident (Figure 1). A detailed description of FETP-Frontline has been published previously [15]. In short, FETP-Frontline includes three 1-week workshops. Through these workshops, trainees learn the components of successful public health surveillance, methods to improve data quality, methods to analyse and interpret surveillance data, and how to investigate cases to assess outbreak potential. Following the workshops, trainees spend one to two months at their usual worksites, where they build and document competencies in disease surveillance through program deliverables such as data quality audits. Thus, trainees are expected to make improvements to the surveillance system by incorporating these program deliverables into their routine work. ZFETP has adapted the standard FETP-Frontline curriculum to meet the changing needs of the MoH. For example, in 2015, UNZA requested that ZFETP enroll ZFETP-Advanced candidates with more experience in data analysis, in order to thrive in the academic rigor of the UNZA MSc program. To meet this need, ZFETP added one week of training in Epi-Info, plus leadership and management skills to the standard FETP-Frontline curriculum for Frontline Cohort 1. ZFETP has also demonstrated flexibility during public health emergencies (or public health events). For example, in 2017 (Frontline Cohort 2), the Minister of Health requested that the ZFETP-Frontline training be extended by two weeks so that trainees could apply their new skills in outbreak investigation and response to support a large cholera outbreak response in Lusaka.

#### Zambia National Public Health Institute

In 2015, the MoH committed to establish the Zambia National Public Health Institute (ZNPHI) as a national public health center of excellence for public health security. The main mandate of the ZNPHI is to improve the health of the people through disease prevention, surveillance and disease intelligence, early detection and response to outbreaks, and health security workforce development. Around the world, NPHIs are focal points for countries´ public health activities, overseeing public health functions that may have previously been spread across multiple entities [6, 7]. A functional NPHI strengthens public health functions through better coordination of programs and activities. NPHIs can support and sustain investments in health security by helping countries strengthen sustainable public health competencies and achieve IHR compliance, as well as serve as the functional homes for health security activities. In Zambia, ZNPHI consolidates the core public health functions of surveillance, outbreak investigation, public health emergency management, information systems, laboratory networks, public health research, and workforce development into one institute [16]. In 2016, all disease surveillance activities moved to the ZNPHI; this change included ZFETP, which moved into the ZNPHI´s Workforce Development (WFD) pillar. Since then, ZFETP is now documented within the official GRZ organizational structure, bringing greater visibility to the program across the government and with partners. Residents participate in high-level strategic meetings and trainings that take place at the ZNPHI, and enjoy strong connections to the Africa Centres for Disease Control and Prevention (Africa CDC), which is a technical institution of the African Union that aims to strengthen national capacities to detect and respond quickly and effectively to disease threats [17]. Under Africa CDC´s operating model, Zambia serves as the Regional Collaborating Center (RCC) for the Southern Africa region; ZNPHI currently hosts the Southern Africa RCC. Bringing ZFETP under the ZNPHI positioned ZFETP to achieve greater impact. Because ZNPHI also houses the public health emergency operations center (PHEOC) and has responsibility for outbreak investigations, ZFETP-Advanced residents are natural first responders to public health events at the national level. Additionally, with ready access to and analysis of national surveillance data, ZFETP residents are well placed to detect potential outbreaks early. ZFETP residents and graduates are key members of outbreak response teams, and the incident management structure of the PHEOC (when activated). Following the completion of a response, residents work with the ZNPHI Information Unit to rapidly disseminate findings in the quarterly public health bulletin, The Health Press, which is also housed within ZNPHI.

#### Partners

ZFETP receives financial and technical support from GRZ, and from multiple partners. GRZ has provided financial support since program inception. Initial support was primarily in-kind, such as paying residents´ salaries, providing meeting spaces, and providing a pool of public health professionals who could serve as field mentors. Recent support has expanded to include supporting a portion of the Frontline workshops and travel for outbreak investigations. To date, the largest financial contributor has been U.S. CDC, with funds primarily from the U.S. President´s Emergency Plan for AIDS Relief (PEPFAR) and the U.S. President´s Malaria Initiative (PMI). In 2018, the United Kingdom´s Department for International Development (DFID) began providing financial support for ZFETP-Frontline. ZFETP is linked to other FETPs as a member country of the African Field Epidemiology Network (AFENET) and Training Programs in Epidemiology and Public Health Interventions Network (TEPHINET). A future goal of the ZFETP-Advanced is to achieve TEPHINET accreditation, which will require a commitment to continuous quality improvement and full institutionalization, both critical for sustainability. Furthermore, the ZNPHI is a member of the International Association of National Public Health Institutes, which has worked with CDC and other partners to strengthen the ZNPHI as Zambia´s premier public health institute [18].

### Program highlights

#### HIV

In Zambia, HIV remains the leading cause of death [19]. Government entities like the National HIV/AIDS/STI/TB Council (NAC) value the importance of strengthening the public health workforce in order to analyse routine HIV surveillance data from government health information systems [20]. ZFETP residents´ work in HIV exemplifies the service component of FETPs, whereby residents learn while providing service to MoH by tackling key HIV issues during the completion of their competencies. For example, in fulfilment of their surveillance evaluation competency, ZFETP residents have identified gaps in case-finding among infants born to HIV-positive mothers. In fulfilment of their planned epidemiologic study competency, residents have described the patterns of HIV infection in female sex workers, explored the role of older men who exchange gifts and money for sex with younger women, identified factors associated with low uptake of HIV testing and treatment in young men (who have the lowest testing and treatment rates in Zambia), and analysed factors associated with poor viral load suppression.

#### Malaria

In Zambia, malaria is endemic with nearly 16 million people at risk of being infected with *P. falciparum*, and a population incidence of 173 per 1,000 persons [21, 22]. PMI is making long-term investments in public health workforce development by supporting one or two residents in each Advanced cohort. These PMI-supported residents are assigned to the National Malaria Elimination Centre (NMEC), where they receive malaria-specific training, and in turn they support NMEC´s goal of ending malaria by undertaking priority projects. They have improved malaria surveillance by evaluating the existing surveillance system and examining ways to improve the reporting of malaria data from private facilities to the MOH´s Integrated Disease Surveillance and Response (IDSR) system; investigated clusters and outbreaks of malaria; investigated knowledge, attitudes, and practices towards malaria prevention in high-risk districts; and investigated factors associated with health-seeking behaviours among parents or guardians with febrile children under age five [23, 24].

#### Outbreak response

One hallmark of the ZFETP is its contribution to outbreak response in Zambia, including recurring outbreaks such as typhoid fever and anthrax, in addition to less-well documented outbreaks such as konzo, (a neurological disease characterized by abrupt onset of an irreversible, non-progressive, and symmetrical spastic para or tetraparesis that is associated with cassava poisoning) [25]. Residents, as part of these investigations, have performed descriptive analyses and conducted case-control studies in order to make disease control policy recommendations based on epidemiologic evidence. As a result, outbreaks are investigated more often and more thoroughly, resulting in numerous publications, and presentations at national and international scientific symposiums on a wide variety of diseases such as meningococcal meningitis, tungiasis, cholera, foodbore diseases, typhoid fever, mumps, and bubonic plague [25-34]. ZFETP´s response to a large cholera outbreak in Lusaka exemplified ZFETP´s impact on outbreak response. During October 2017 - March 2018, the MoH recorded an estimated 5,900 cholera cases and 114 fatalities. ZFETP residents described the affected areas and highlighted gaps in safe water and sanitation: only one-half of households understood that cholera was transmitted via contaminated water, and only one-third of households were drinking water with adequate levels of chlorine. The residents then conducted a case-control study to investigate risk factors for morbidity, and found that cases had increased odds of drinking borehole (i.e. well) water [28]. Based on this information, GRZ installed emergency water tanks that contained chlorinated water, and the outbreak came to an end. During this outbreak, residents also staffed the public health emergency operation centre (PHEOC). They conducted cholera vulnerability risk assessment and mapping, and generated evidence-based research for decision making. They also participated in oral cholera vaccination campaigns, as well as disease sensitization and community health education.

### Challenges

#### Reliance on donor support

to date, the ZFETP has relied on outside funding for most program costs. This reliance on external funding poses risks to the program, especially if that support was to suddenly dissipate. To mitigate this risk and to chart a path towards greater institutionalization and sustainability, GRZ has begun to increase domestic financing for ZFETP. The government has committed to supporting FETP expansion, as evidenced through the creation of a budget line in the 2019 national approved budget for expenditure [35]. Additionally, the National Action Plan for Health Security will be launched by the President of Zambia; again, showing the high-level political will to strengthen health security in the country. In addition, ZNPHI has engaged with partners to diversify funding sources.

***Limited human resources:*** ZFETP´s long-term staffing strategy includes several permanent positions: (1) a program manager, who provides overall leadership and decision-making, sets strategic direction, and advocates for resources; (2) a program administrator, who provides day-to-day operational oversight and manages program finances and logistics; (3) an FETP-Advanced program coordinator, who oversees Advanced recruitment and activities and coordinates mentors; (4) a FETP-Frontline program coordinator, who oversees Frontline recruitment and workshops and coordinates mentors; and (5) a driver. Currently, only two of these positions (program manager and administrator) are filled, and both are dependent on U.S. CDC funds. As of the time of writing, the GRZ intends to formally fund the full structure of staff at the ZNPHI once Parliament signs the ZNPHI bill into law. This will allow ZNPHI to hire additional staff to meet the ZFETP´s staffing needs.

***Inadequate mentorship:*** ZFETP seeks to use advanced graduates to mentor current residents, both in FETP-Frontline and FETP-Advanced. However, because the program is new, the number of graduates (i.e. potential mentors) is low. Mentoring is not included in the standard MoH job description; thus, even the best mentors find it difficult to create time to work with residents. Finally, FETP-Advanced graduates are not distributed evenly across the country; half remained in the capital city following training, and one province has no advanced graduates. Thus, mentoring residents and trainees in hard-to-reach areas of the country can be challenging and resource-intensive. As ZFETP graduates additional advanced cohorts, the number of qualified mentors will grow, and this will help to alleviate the current mentoring gap. In the meantime, a newly formed Alumni Forum will provide a networking platform for all ZFETP alumni to mentor and collaborate, and to serve as a pool of potential responders for outbreaks. Additionally, ZFETP plans to keep the momentum of graduates as mentors through a variety of innovations such as revising MoH work plans to include mentoring in job descriptions, holding mentor-specific workshops, handing out awards to recognize superior mentoring, and providing opportunities for mentors to travel to conferences where their mentee´s work has been accepted. Further, discussions are underway to establish positions for field epidemiologist within the MoH and ZNPHI structures; a move that will clearly define career paths for the graduates.

## Conclusion

The Zambia FETP has been successful in responding to public health concerns and emergencies, such as high-profile outbreak investigations, and these epidemiologic investigations have contributed to the body of research in Zambia. Housing the program within the ZNPHI, which oversees national disease surveillance and outbreak response, has increased ZFETPs visibility and catalyzed its impact and institutionalization. Within a relatively short time, the ZFETP has made clear contributions to national health security. The next 5 years will be important to continue the program´s path toward full institutionalization and sustainability, and to implement continuous quality improvement efforts to address the challenges which face all new FETPs [12]. Along these lines, ZFETP plans to develop and implement a plan to routinely evaluate both the Frontline and Advanced programs, in order to determine areas of success as well as areas in potential need of improvement.
